# Two birds with one stone: Doing metabolomics with your proteomics kit

**DOI:** 10.1002/pmic.201300192

**Published:** 2013-11-21

**Authors:** Roman Fischer, Paul Bowness, Benedikt M Kessler

**Affiliations:** 1 Target Discovery Institute, Nuffield Department of Medicine, University of OxfordOxford, UK; 2 Nuffield Department of Orthopaedics, Rheumatology and Musculoskeletal Sciences, University of OxfordOxford, UK

**Keywords:** Integration, Liquid chromatography, Mass spectrometry, Metabolomics, Technology

## Abstract

Proteomic research facilities and laboratories are facing increasing demands for the integration of biological data from multiple ‘-OMICS’ approaches. The aim to fully understand biological processes requires the integrated study of genomes, proteomes and metabolomes. While genomic and proteomic workflows are different, the study of the metabolome overlaps significantly with the latter, both in instrumentation and methodology. However, chemical diversity complicates an easy and direct access to the metabolome by mass spectrometry (MS). The present review provides an introduction into metabolomics workflows from the viewpoint of proteomic researchers. We compare the physicochemical properties of proteins and peptides with metabolites/small molecules to establish principle differences between these analyte classes based on human data. We highlight the implications this may have on sample preparation, separation, ionisation, detection and data analysis. We argue that a typical proteomic workflow (nLC-MS) can be exploited for the detection of a number of aliphatic and aromatic metabolites, including fatty acids, lipids, prostaglandins, di/tripeptides, steroids and vitamins, thereby providing a straightforward entry point for metabolomics-based studies. Limitations and requirements are discussed as well as extensions to the LC-MS workflow to expand the range of detectable molecular classes without investing in dedicated instrumentation such as GC-MS, CE-MS or NMR.

## 1 Introduction

The use of MS has become an essential part in today's biological and biomedical sciences. MS is particularly powerful when combined with LC-based separation of the analyte, and has now become one of the most commonly used techniques to detect a large number of accessible biomolecules 1. Proteomics is strongly associated with the extensive use of MS and focuses on the qualitative and quantitative analysis of proteins, peptides and their PTMs. While in basic research the use of proteomic workflows has generated immense knowledge about biological processes and disease mechanisms, the implementation of proteomic markers for the detection and prediction of diseases 2,3 is lacking, despite significant financial investment. More recently, clinical scientists searching for molecular markers are turning towards the metabolome, as the analysis of small molecules in patient-derived samples such as blood and urine promise an instantaneous snapshot of the subject's physiology. At the same time, laboratories focused on proteomics are facing a growing demand for integrative studies, starting from a systematic analysis of genome versus proteome comparisons and more recently for correlative studies between the metabolome, the genome and proteome 4. Genomic data sets are acquired with entirely different equipment, and their integration with proteomics results requires intense interactions between specialised laboratories. Metabolites are traditionally studied by analytical chemists using NMR, GC-MS, LC-MS and CE-MS while most proteomic researchers have a strong background in biophysics, chemistry or biochemistry and preferentially use nLC-MS-based workflows.

A major hurdle in metabolomics-based studies remains the limited characterisation of the human metabolome. While the genome and the proteome are now well annotated and defined by the genetic code, the metabolome has fewer fundamental restrictions. The metabolome is defined as the entirety of molecules processed by the metabolism in an organism. The vast majority of metabolites have a mass below 1500 Da (Fig.2A) but especially lipids can be observed with higher masses up to 5000 Da 5. From a chemical/analytical point of view, the metabolome needs to be divided into sub-metabolomes (i.e. sugars, lipids, nucleotides, amino acids, etc., see Table2) according to their chemical properties. However, the classification of the molecular diversity is challenging 6. Also there is no solid line separating metabolome and proteome, exemplified by the ‘peptidome’ (∼0.4–12 kDa), usually degradation-derived short protein fragments, which have been observed to have multiple biological functions such as bone turnover or regulation of blood pressure and inflammatory response (reviewed in 7–9). Shorter di- and tripeptides have been observed to have biological functions in the protection against oxidative stress and immune deficiency (i.e. GSH) or can have antiviral activity 10.

**Figure 1:**
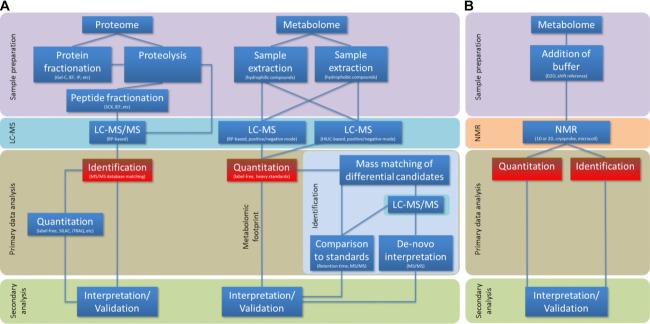
Conceptual differences between a typical proteomics workflow and possible metabolomics workflows. (A) A ‘shotgun’ proteomic discovery experiment will typically employ a pre-fractionation of the analyte pre- or post-proteolysis, followed by LC-MS/MS analysis. Identification of peptides/proteins is essential for both quantitation and interpretation. A metabolomic experiment requires a sample extraction compatible with the analytical workflow further downstream. A separation into hydrophilic and hydrophobic compounds (Supporting Information Fig. 2) can yield samples for HILIC and RP front-end separation. The quantitation of detected molecules builds the basis for further processing. Even without identification, a metabolomic footprint can be used for diagnostic purposes and differential analyses. (B) The NMR-based metabolite sample preparation and analysis is not limited towards compounds with physicochemical properties compatible with LC-MS. Minimal to no sample preparation is needed. However, NMR (as other powerful platforms for metabolomics such as GC-MS or TLC-GC-FID 115) is not a standard technique used in most proteomics laboratories and is considered less sensitive than MS.

**Figure 2:**
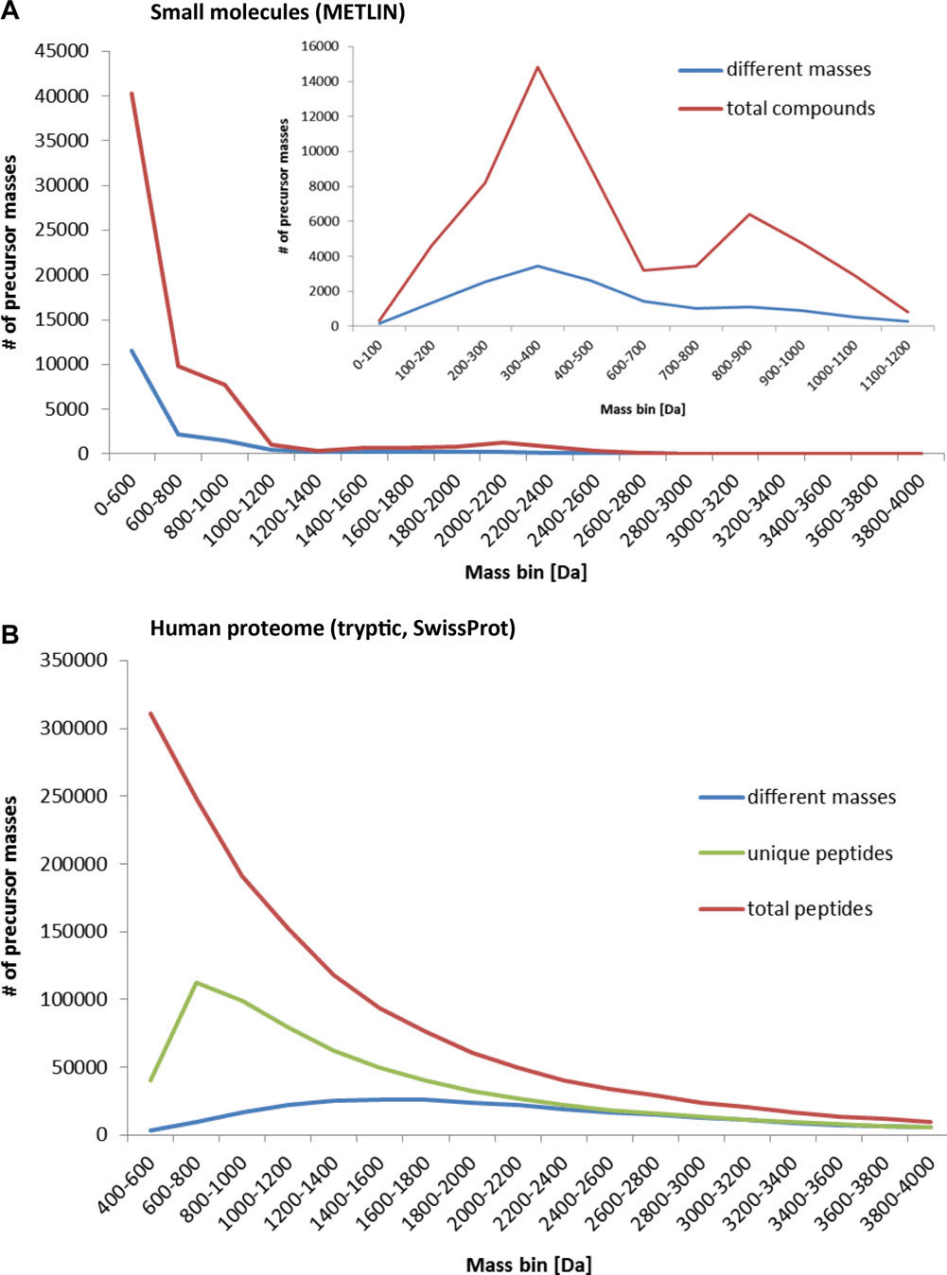
Mass redundancy of biomolecules – a challenge for identification by MS. (A) All 64 092 molecular entries of the METLIN database were sorted based on their molecular masses and then categorised in mass bins of 200 Da (X-axis). The total number of compounds per mass bin (red line) and the number of different masses (blue line) are displayed, indicating an uneven distribution of compounds and those sharing identical masses across the mass range (insert: higher resolved plot for mass range 0–1200 Da). (B) All protein entries from the SwissProt (UniKProt, 21 March 2012 release, containing 35 956 unique proteins incl. isoforms) database were digested in silico with trypsin (Protein Digestion Simulator by Matthew Monroe, PNNL (USA)) yielding 1 501 402 protein fragments between 400 and 4000 Da, sorted based on their molecular masses and then categorised in mass bins of 200 Da (X-axis). The total number of peptides per mass bin (red line), the number of unique peptides (green line) and the number of different masses (blue line) are displayed, indicating an uneven distribution of total and peptides with different molecular weights across the mass range.

The analytical problem arising from the chemical diversity of the metabolome is immense. The consequence of the chemical diversity in the metabolome of an organism is that researchers frequently study one sub-metabolome with one analytical workflow at a time, perhaps tailoring the analysis method to the compound of interest. For example, expertise and methodology may concentrate on specific metabolites such as the ‘lipidome’ rather than metabolomics as a whole. By contrast, a broader ‘-OMICS’ approach (which aims to study the metabolome of an organism) will employ a variety of complementary (bio)-chemical extraction, separation and analytical methods. Therefore, an ‘-OMICS’ approach in the context of small molecules is potentially even more challenging to perform than in proteomic or genomic research – even when protein modifications or epigenetic variations are considered.

To meet the vast variety of chemical properties of metabolite classes 11, a comprehensive analysis of the metabolome requires different separation and ionization methodologies – such as GC-MS, LC-MS, CE-MS 12, ESI, atmospheric pressure chemical ionization (APCI), FAB and MALDI 13. In addition, NMR spectroscopy has offered an alternative measurement strategy for metabolites 14. The advantages of NMR analysis of metabolite samples are the non-destructive nature of the method and the detection of compounds independent of their molecule class. Other advantages comprise extremely simple (automatic) sample preparation, short acquisition times and high reproducibility and robustness. The sensitivity of NMR is in general lower than MS with detection limits in the nanomol range while modern mass spectrometers can detect compounds in the low attomol range. An extensive comparison between NMR- and MS-based methodologies for metabolomics analysis has been reviewed elsewhere 15.

GC-MS has long been the established method for measuring volatile metabolites and compounds 16. More recently, CE-MS and LC-MS have emerged as suitable alternatives, LC-MS being the most versatile methodology capable of separating and detecting the greatest portion of metabolites 1,17,18. Different LC chemistries such as hydrophilic interaction chromatography (HILIC) 19, RP 20, but also ion-exchange 21 and monolithic solid phases 22 have been developed and combined with MS for mass detection. RP LC, predominantly based on C18 silica beads using an acidic water/organic mobile phase combined with ESI, has gained wide popularity in MS and proteomics laboratories, as this combination appears to be suitable for the separation of peptides based on their biochemical diversity, in particular in the nano-flow mode 23 (Table1). In tryptic digests the C-terminal basic residue (lys/arg) allows a facile protonation under acidic conditions in positive ESI mode. This setup can not only be exploited for the detection of peptides and proteins, but also for a number of aliphatic and aromatic metabolites, such as fatty acids, lipids, prostaglandins, di/tripeptides, steroids, vitamins 24 and nucleic acids 25 (Table2). Neutral or basic mobile phases in combination with negative mode ESI offer the detection of negatively charged aliphatic compounds, but negative ion formation is less efficient due to the use of nonpolar solvents, the occurrence of electrical discharge (noise) and reduced solvent desolvation. Nevertheless, LC-ESI-MS seems to represent a suitable entry point for proteomics specialists into metabolomics and the analysis of small molecules 1.

**Table 1:** Common requirements for various aspects of proteomic and metabolomic sample analysis

	Proteomics	LC-MS based metabolomics
MS instrumentation	Ion trap, Q-TOF, QqQ, hybrid, Orbitrap	TOF, Q-TOF, QqQ, single-quad, Orbitrap
Ionisation	ESI, MALDI	ESI, APCI
Detector	MCP, electron multiplier, Orbitrap	MCP, electron multiplier, Orbitrap
Polarity	Positive	Positive/negative
High resolution	Required	Optional
High mass accuracy (MS1)	As high as possible	70 ppm or better
High scan speed	Required	Optional
High sensitivity	Required	Required/less critical
High dynamic range	Required	Required
MSn capability	Required	Optional (comparison to standards)
High mass accuracy (MSn)	Optional	Required
High resolution	Optional	Required
Chromatographic separation	Required	Required (screening)
Column chemistry	RP (HILIC)	RP, HILIC, others
Nano-flow	Required	Normal flow preferred
Injection volume	0.5–10 μL	1–100 μL
Long columns/gradients	Required	Optional
Low inter-day variability	Optional	Required
Software for analysis	Vendor-specific, commercial or free software	Vendor-specific and limited free software
MSMS analysis	Automated	Manual
Databases	Available for sequenced organisms	Incomplete
Identification of analyte	Required	Optional
Use of standards	Optional (absolute quantitation, SWATH)	Required

**Table 2:** Accessibility of selected metabolite classes using reversed and HILIC stationary phases

Compound class	C18	HILIC
Acyl glycines	62	63
Amino acids	64	65
Amino alcohols	64	64
Bile acids	66,67	
Biotin and derivatives	64	64
Carbohydrates		68
Carnitines		69
Catecholamines and derivatives		70
Cobalamin derivatives	71	
Coenzyme A derivatives	64	64
Dicarboxylic acids	72	73
Fatty acids	74	75
Glucuronides	76	77
Glycerolipids	78	79
Hydroxy acids	80	81
Indoles and indole derivatives	82	
Keto acids	83,84	81
Leukotrienes	85	
Lipoamides and derivatives	86	
Nucleosides		87
Nucleotides		65
Peptides	88	89
Phospholipids	90	91
Polyamines		92
Polyphenols	93	
Porphyrins	94	
Prostanoids	95	
Pterins	96	97
Purines and purine derivatives	64	64
Pyridoxals and derivatives		98
Pyrimidines and pyrimidine derivatives		87
Retinoids	99	
Sphingolipids	100	101
Steroids and steroid derivatives	102	
Sugar phosphates		103
Tricarboxylic acids		104

Given the similarities in instrumentation and analytical workflows, there is surprisingly little integration between researchers of both disciplines metabolomics and proteomics. Nevertheless, the complete understanding of biological processes often suffers from such an analytical segregation as it usually involves the interaction of proteins and small molecules. While the integration of genomic and proteomic data is driven forward by both disciplines, researchers are only now beginning to develop tools to examine perturbations in the proteome, genome and metabolome in order to develop a holistic view on biological processes and disease mechanisms.

This review, addressed primarily to the proteomic researcher, outlines ways to explore the analysis of small molecule compounds that are compatible with equipment used for proteomics (Fig.1, Table1). We compare the proteome with the metabolome from a technical/analytical viewpoint to illustrate the limitations but also the opportunities the proteomic researcher may face when complying with an increasing demand in metabolome research. Even though we discuss some of the specific instrumentation used in metabolomics analysis, we emphasise that most proteomics laboratories already have the capability to analyse metabolome samples with minimal investment into new equipment and expertise (Table1). We outline the challenges and difficulties that a proteome researcher may be confronted with when embracing and adapting existing methods and instrumentation for metabolomics studies, and provide a basis for discussion about realistic expectations for metabolite studies in proteomics labs.

## 2 Proteins/peptides versus metabolites

The proteome in higher organisms is complex and highly dynamic. While certain proteins are only expressed in a specific biological context, other proteins become modified post-translationally as a result of signalling events. Another layer of complexity is added by changes in subcellular localization or the overall function of the analysed cell-type in an organism. In humans, the complete proteome consists of 20 248 reviewed, unique proteins (Uniprot, 21 March 2012 release) or 35 956 proteins including isoforms. Their masses range from 1.419 kDa for the protein LST1 to 2.99 MDa for Titin 26.

The metabolome as an analyte is challenging as there is no underlying genetic code from which the chemical composition of a metabolite can be deduced. Consequently, most available knowledge in metabolite databases is based on experimental observations. The diversity of current databases covering metabolites of plants, animals, drugs of different sources is far more complex than available data for the human metabolome (http://www.metabolomicssociety.org/database). Also, the databases covering human metabolites are highly segregated (http://www.biomedcentral.com/1752-0509/5/165) and most likely not comprehensive. While 41 519 metabolites have been described in version 3.5 of the Human Metabolome Database 27,28, researchers are also confronted with secondary metabolites, endogenous peptides and exogenous metabolites including drugs and their degradation products when analysing primary samples. A fairly comprehensive database including some of these confounding compounds is the METLIN database, which currently comprises 64 092 entries between 16 and 4723 Da (as of 2013 5). To illustrate the analytical challenge that the metabolome (METLIN) and the proteome (SwissProt) provide, the number of precursor masses was plotted against mass bins (Fig.2). The 64 092 chemically unique compounds in the METLIN database exhibit 17 058 different masses, and 8828 compounds have unique masses (Supporting Information Fig. 1) when the probable formation of adducts, multimers and multiple charge states during ionization are omitted for clarity. The high mass redundancy can be explained by the existence of many stereoisomers, enantiomers, etc. and redundancy in compound classes such as tripeptides (300–400 Da) and lipids (300–400 Da and 1000–1100 Da). Consequently, different chemical formulae not infrequently have identical atomic composition and molecular weight (Fig.2A and insert).

Similar analysis of the human proteome reveals that on the protein level, 35 919 masses in 35 956 proteins are different and 35 896 proteins – when considered unmodified – could be identified by this property only (Supporting Information Fig. 1), providing that certain technical limitations could be overcome. In an acidic milieu, proteins exist with multiple positive charge states. As mass spectrometers detect mass to charge ratios, a single protein will be detected as multiple entities (charge state envelopes). Each of those entities will also have an isotopic pattern according to the presence of natural stable isotopes of carbon and nitrogen and to a lesser extent of sulphur and oxygen. To determine the charge state and ultimately the mass of a protein, the isotope-derived signals need to be resolved by the mass spectrometer. Intact proteins are usually observed at *m*/*z* ratios between 800 and 5000 Da exhibiting charge states of 50 and higher. Orbitrap 29 and FT-ICR mass spectrometers, achieving a resolving power of 240 000 and higher 30,31 at *m*/*z* 400, are able to resolve the charge state of smaller proteins. However, this type of analysis usually requires a pure sample and is currently not considered routine or suitable for high throughput 32. If the charge state cannot be resolved, the average protein mass can be calculated within low ppm mass accuracy after deconvolution of the differentially charged entities.

Modifications such as phosphorylation, acetylation or oxidation commonly occur in proteins, multiplying the number of observable masses, which complicates the identification of a protein solely by its mass. Additional fragmentation data are therefore often necessary to determine the C- and N-terminal sequence of a protein for its identification by sequence tags and de novo sequencing, currently achievable to some extent on intact proteins using electron transfer dissociation technology 33. Even though it is tempting to utilise the principal uniqueness of a protein's mass for its identification, mass spectrometers and data analysis still have to evolve to fulfil the technological requirements when analysing complex protein mixtures.

To bypass the above-mentioned difficulties and for the benefit of more accurate and sensitive analysis by MS 26, proteins are proteolytically cleaved for most analyses, breaking the proteome down into much more complex enzyme-specific peptide pools (shotgun proteomics and PMF). We used Protein Digestion Simulator v2.2.3992.29199 by Matthew Monroe (http://omics.pnl.gov/software/ProteinDigestionSimulator.php) to generate an in silico digest of the human proteome with the commonly used proteolytic enzyme trypsin. This resulted in a total of 1 501 402 protein fragments in the MS-relevant mass range of 400–4000 Da. Because of homologous amino acid sequences in different proteins or isomers, only 43.5% (653 698) of these peptides have a unique sequence. However, peptides of different sequences can have the same amino acid composition and therefore the same exact mass, resulting in 279 002 (18.6%) different masses of which 197 709 are unique, so the determination of the peptide mass alone would be sufficient for the identification of these peptides (Supporting Information Fig. 1). Our calculations have their limitations – for example they do not consider the presence of PTMs – although in most cases, the unmodified peptide is also observed. In addition, these calculations also ignore the problem of missed cleavage sites in enzyme-generated peptide pools. However, the occurrence of missed cleavage sites 34 can be predicted with high sensitivity and specificity by the algorithm iSpider (http://ispider.smith.man.ac.uk/MissedCleave/) considering extended cleavage rules for trypsin. Since a missed cleavage in a complete digest of the proteome would increase the length of a tryptic peptide, the number of different peptide species in a real tryptic digest of the human proteome is likely to be lower than estimated in our simplified calculations. Trypsin is the most commonly used proteolytic enzyme in proteomic workflows due to its specificity, availability and the tendency to generate protein fragments that are suitable for mass spectrometric analysis (positive charges both at the N-terminus and basic C-terminal residue, which facilitates ionization under acidic conditions in positive mode MS). However, other enzymes such as chymotrypsin, Lys-C, Glu-C (V8), Asp-N or elastase can also be used to generate alternative cleavage products. Therefore, we conducted a similar analysis for Arg-C, Asp-N, Glu-C and Lys-C, which is available as Supporting Information (Supporting Information Fig. 3). With the exception of Glu-C, the proteases alternative to trypsin generate longer peptides and therefore less mass redundancy and less complex samples (Supporting Information Tables 1 and 2). In conclusion, both proteomic and metabolomic analytes represent a considerable challenge for the analysis by MS, but not necessarily for the same reasons.

From an analytical viewpoint, the digested proteome displays extreme complexity with at the same time high chemical uniformity of its entities, even when PTMs are considered. The metabolome on the other hand features a large chemical diversity with a less complex compound composition, especially after sample preparation/enrichment. As a consequence of the high chemical diversity, only metabolic molecules with similar physicochemical properties such as peptides can be separated and detected by (n)LC-MS: Khanna and Ranganathan 35 described the property space distribution among human metabolites and predicted that only ∼17% of the metabolites in the human metabolome database (6582 entries in 2008) have a negative n-octanol/water partition coefficient (ALOG P) and are therefore water soluble and accessible to LC (LC-MS) methods. Besides the solubility in water, another relevant parameter for the detection of a small molecule by nLC-MS approaches is the ionisation efficiency. While the ionisation efficiency can be predicted using ALOG P, molecular volume and effective charge of a molecule 36, for the estimation made above, we postulate that the capability of the chromatographic system to provide different ion pairing agents and also to reduce ion suppression allows the ionisation of the majority of the water-soluble compounds.

## 3 Sample preparation

Both metabolomics and proteomics approaches can use a wide range of sample materials ranging from body fluids to cellular extracts and tissue culture supernatants. Sample collection and variability are equally important in both fields and the stability of a sample can be a major concern, particularly for metabolomic analysis. While proteins (once denatured and in presence of protease inhibitors) are relatively stable, they can gain or lose PTMs after prolonged storage. Nevertheless, if this occurs their peptides still can be identified taking the modifications (i.e. oxidation, deamidation or dephosphorylation) into account. Metabolites however will degrade into other compounds, potentially rendering the direct detection or identification of the precursor metabolite impossible. Therefore, although samples appear relatively stable for the first 2 h 37, sample collection and storage needs to be highly consistent to avoid the introduction of variability by differential degradation between samples. MS-based detection methods usually require sample preparation, which is guided by the analytical workflow and properties of the compatible compound classes, which makes metabolite samples susceptible to degradation. Gika et al. 38 demonstrated for urine that midterm sample stability can be archived by storage at −20°C and observed no changes in the sample for up to nine freeze-thaw cycles while sample degradation became evident after 48 h at 4°C. However, the sample preparation and storage conditions need to be tailored for the compound class and sample type as exposure to active enzymes, reagents or prolonged dwell times during sampling may lead to degradation 39,40. This problem is aggravated by the different dwell times of samples in the LC-MS autosampler when analysing a large number of metabolite samples. It is therefore advisable to track changes in the sample composition in addition to address the stability of the analytical workflow (drifts in mass detection and chromatographic separation). We support analysing a quality control standard consisting of the mixture of all samples before, during and after the analysis of the metabolite samples 41. This quality control standard should be analysed several times before the injection of real samples to equilibrate the analytical workflow to the specificities of the sample type (matrix effect 42). The quality control can be used to evaluate the stability of the system and sample carry-over. This is especially important, as in metabolomics the analyte is usually singly charged and can more easily be mistaken for a contamination – a problem that is omitted in proteomics as peptides are usually observed with multiple charges. It is also recommended to randomise samples or even technical replicates to avoid the introduction of systematic errors due to degradation processes or carry-over between samples.

The sample preparation for a proteomic (shotgun) experiment has evolved from a gel-based separation of proteins according to one or two chemical properties (mostly size and p*I*) 43, followed by proteolytic cleavage while still residing in the gel matrix. The in-gel digestion methodology is nowadays the preferred approach as compared to electro-elution of protein material into solution or onto nitrocellulose/PVDF membranes, as the recovery efficiencies of these techniques are relatively poor. Peptides are then extracted and purified before MS analysis. Usually proteins are precipitated to eliminate compounds that would interfere with the proteolytic enzyme of choice after or before increasing the accessibility of proteolytic cleavage sites by denaturing the proteins and blocking the otherwise highly reactive cysteine residues. If re-solubilisation of the protein of interest is crucial, desalting columns, dialysis or ultra-filtration can be employed to purify the protein (intact protein MS). The proteolytic digest usually takes several hours and is followed by a desalting step to purify and concentrate the resulting peptides. Altered strategies to increase the speed 44, efficiency of proteolytic digest 45 and sequence coverage 46,47 have been widely explored. However, they represent extensions of current practise rather than novel procedures. Even though proteomic sample preparation is a standard technique in many laboratories 48, it can prove to be a challenge even for experienced proteomics facilities 49. Sample contaminations with polymers or keratin are relatively common and can be challenging to eliminate. More sophisticated sample preparation techniques such as protein pre-fractionation (i.e. subcellular fractionation, IEF and SEC) or PTM enrichment are rarely performed outside a proteomics laboratory.

While the principles of proteomic sample preparation are fairly well established, the preparation of a ‘metabolomics’ sample strictly depends on the compound class the researcher is interested in 18. Integrative projects involving proteomic and metabolomic analyses seek to detect compounds that change their abundance between sets of different biological samples. Therefore, metabolite analysis (as well as proteome analysis) often contains a quantitative component, making robust and simple sample preparation essential. Metabolite extraction can be achieved by liquid–liquid extraction, solid–liquid extraction or SPE (i.e. HILIC, SCX, WAX, C8, etc.). The extraction method used defines the composition of the extracted metabolites and should be designed according to the used analytical workflow. The chromatographic separation method of choice in proteomic laboratories is RP chromatography (RP-LC), which resolves water-soluble peptides according to their hydrophobicity as a function of amino acid composition and peptide length. The same chromatographic principles separate only a subgroup of metabolites with similar hydrophobicity as peptides. Besides the sometimes successful ‘Dilute and Shoot’ method 50, a good starting point for metabolite extraction is a precipitation method, which is widely used in both protein and metabolite sample preparation, the chloroform–methanol extraction 51. While the chloroform fraction would be suitable for an analysis with a GC-MS or HILIC-based LC-MS workflow, the aqueous phase can be dried and is then compatible with RP and normal-phase chromatography (Supporting Information Fig. 2). However, there is no standardised method for metabolite sample preparation and the isolation of metabolites requires different SPE protocols guided by the compound class the researcher is interested in 52. Alternative sample preparation methods very often are differing from the standard RP separation used in a proteomics laboratory 53,54.

Next to GC-MS, CE-MS and NMR, LC-MS has emerged as a major analytical platform for metabolite analysis. The sensitivity and speed of modern mass spectrometers in combination with ultra (high) performance LC enables the identification of the practically complete active proteome from 100 μg of crude cell extracts 55. HPLCs used in analytical workflows designed for proteomics usually employ nano-flow settings (column ID < 0.1 mm, flow rate < 1 μL/min) to increase peak capacity, chromatographic resolution and sensitivity 56 (see also below). A trade-off is limited sample volume injection and column capacity, and also lower reproducibility. Nano-flow is used whenever sample amount is limited – this is usually the case for proteomic analyses of biological and clinical (tissue) samples. In contrast, many metabolomic projects that focus on urine, blood or other body fluids are less limited in sample material and therefore more compatible with micro-flow chromatography techniques. While both nano- and micro-flow suffer from ion suppression 57, normal flow omits technical problems typically associated with nano-flow such as spray stability in ESI mode, high back pressure or dead volumes. Normal flow appears to be more suitable for high throughput and targeted analyses due to its better chromatographic reproducibility and generally shorter sample analysis times. However, nano-flow has improved sensitivity, making it the chromatography of choice where sensitivity and sample amounts are important (discovery-type experiments).

The intrinsic difficulties of nano-flow in chromatographic separation and metabolite abundance are the reasons for generally higher flow rates in metabolite analysis. However, nano-flow has been applied to metabolite screening of serum 24,58, single-cell metabolome analysis 59 or biofluid spots 60.

Proteomic nLC-MS workflows heavily rely on RP beads as stationary phase, as it provides optimal properties for the chromatographic separation of peptides. Also HILIC columns have been used for peptide separation and can provide a semi-orthogonal separation to RP chromatography. Both stationary phases have been used to detect metabolites of different classes (Table2). Their capabilities can be further extended by altering the conventional water/ACN buffer system. Volatile cationic compounds form ion pairs with negatively charged metabolites to improve retention and separation on the column 61. Ion-pairing methods have been employed to analyse negatively charged metabolites such as nucleotides or sugar phosphates on RP and HILIC phase.

## 4 Ionisation

In the last two decades, API has been established as the major LC bound soft ionization principle in both proteomics and metabolomics research fields. While ESI is the most common technique in proteomic LC-MS instruments, other API variants (APCI and atmospheric pressure photoionization (APPI)) have been employed for nonpolar compounds in metabolomic studies 105. ESI is suitable for the ionization of large biomolecules such as peptides and proteins, and results in molecules acquiring multiple charges during the ionization process in an electrostatic field. APCI is based on the ionization of solvent molecules by electrons discharged by a corona needle. The charge is then transferred to the analyte molecules by chemical reactions resulting in singly charged compounds, which limits its application to smaller and thermally stable biomolecules. The ionisation of analyte molecules in APPI mode is facilitated by an UV lamp, which generates photons with optimised ionization energies. APPI is most useful when analysing less polar compounds such as steroids at micro-flow rates (<100 μL/min). The best ionization method is dependent on the biochemical properties of the analyte molecules and the sample separation conditions used. However, APPI and APCI ion sources are not widely used in proteomics laboratories, so that most proteomic researchers venturing into the metabolomic field will be limited to ESI and nESI sources. If a heated ESI source is available, ionization of some compounds may be improved over non-heated ESI sources 61.

In situ ionization techniques such as desorption ESI (DESI) 106, direct analysis in real time (DART) 107, desorption APPI (DAPPI) 108 or by laser ablation ESI (LAESI) 109 are recent developments, which facilitate the direct analysis of mostly clinical samples. The ionization of the analyte occurs on the sample surface, which enables the direct analysis of body fluids, plant material, tissue or even single cells. The wide range of recently developed ambient ionisation techniques has been reviewed elsewhere 110. While these methods are useful in very specific settings (MS imaging, drug toxicity analysis, etc.), they generally lack sensitivity due to omitting sample purification or enrichment and are barely used in proteomics laboratories.

## 5 Detection

Mass analysers can be separated into two main groups: (i) trapping mass spectrometers such as ion trap (IT) or Orbitrap and (ii) scanning mass spectrometers employing quadrupoles such as TOF detectors 111. Most proteomics laboratories are equipped with an IT, which offers fast scan rates, high sensitivity and multi stage mass spectrometry. This type of instrument is ideal for the analysis of highly complex peptide mixtures and can achieve high proteome coverage. Because of its limited resolution and mass accuracy, IT mass spectrometers are rarely used in metabolomic approaches. On the other hand, quadrupole and TOF instruments have limitations in sensitivity and scan rate. Hybrid instruments have emerged, which balance the advantages and limitations of different instrument types. For instance, triple-quadrupole instruments are still very limited in resolution and mass accuracy, but they offer a high dynamic range and excellent sensitivity, which make them ideal instruments for peptide and small molecule quantitation in targeted analyses. A hybrid of the highly efficient quadrupole and a highly accurate and resolving Orbitrap is ideal for both peptide and small molecule detection/identification. More detailed comparisons of MS instrumentation have been reviewed elsewhere 111. By combining different mass analysers in hybrid mass spectrometers, the instrumentation can be tailored to specific research fields. Nevertheless, most modern instruments used in proteomic workflows can be used for the analysis of small molecules other than peptides. For the analysis of complex peptide samples, most proteomics laboratories use ion (orbi)trap or TOF-based instruments, which exhibit a mass accuracy below 2 ppm and are used with a mass resolution of up to 100 000 in shotgun proteomics analysis 112. Generally in LC-MS-based metabolomics workflows, Q-TOF instruments are used for discovery and triple quadrupole (QqQ) instruments are used for targeted approaches 61.

## 6 Mass accuracy

**Figure 3:**
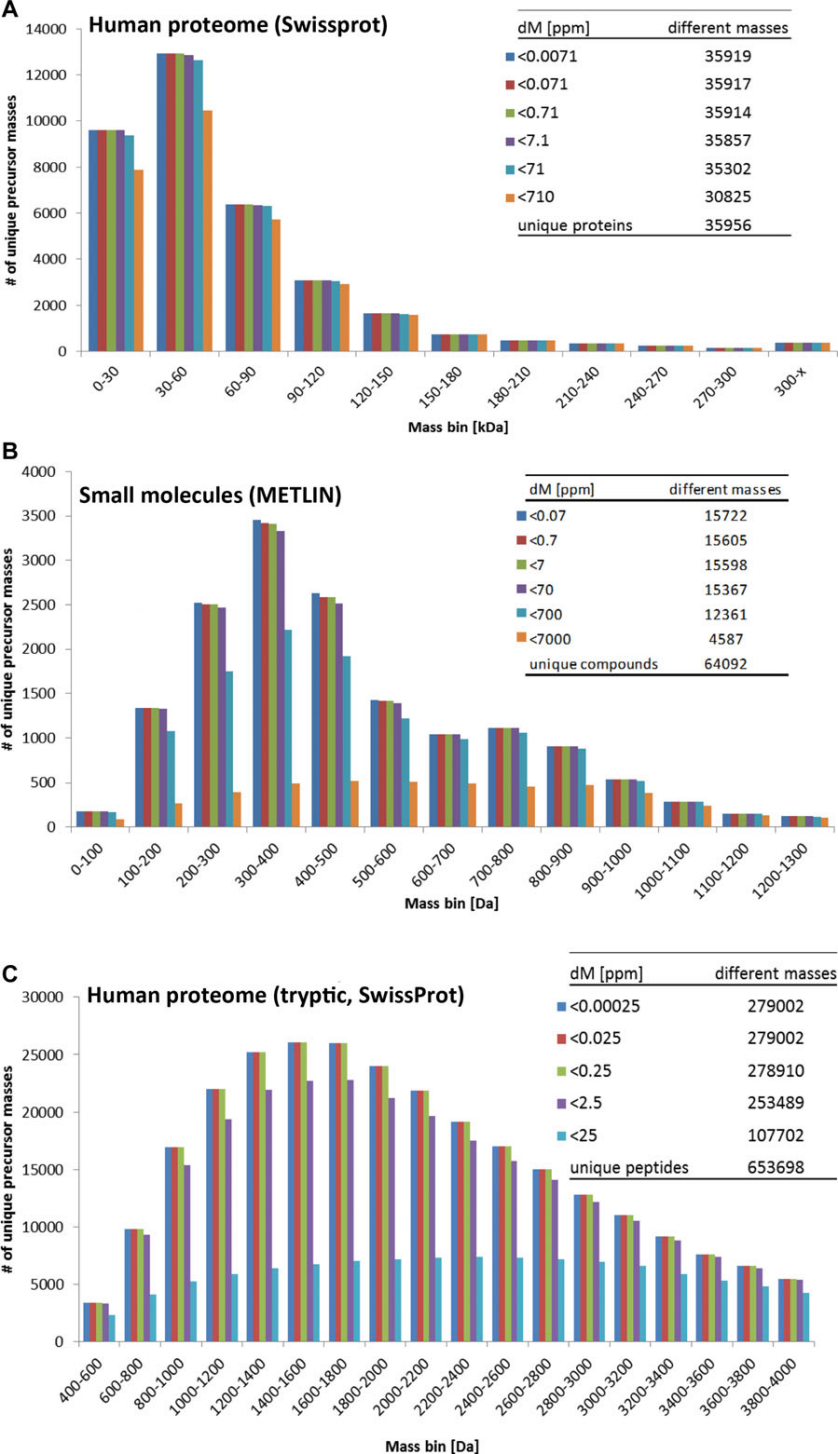
Effect of mass accuracy on measuring and identifying biomolecules. (A) Display of the number of different protein precursor masses present in the SwissProt (UniKProt) database that can be separated based on mass accuracy (ppm calculations are based on protein LST1, 1.419 kDa). The proteins are indicated in groups of 30 kDa mass bins (X-axis). (B) Display of the number of different small molecular compound masses present in the METLIN database that can be separated based on mass accuracy (ppm calculations are based on methane, 16.0313 Da). The compounds are indicated in groups of 100 Da mass bins (X-axis). (C) Display of the number of different peptide precursor masses derived from an in silico trypsin digestion of proteins present in the SwissProt (UniKProt) database that can be separated based on mass accuracy (ppm calculations are based on the unique peptide HNM (Q9C037–3), 400.1528814 Da). The peptides are indicated in groups of 200 Da mass bins (X-axis). A similar analysis was performed after in silico digestion of proteins with other proteolytic enzymes (Supporting Information Fig. 3 and Supporting Information Tables 1 and 2).

As pointed out earlier, mass redundancy on the peptide and metabolite level remains a challenge for compound identification. While there is no doubt about the importance of high mass resolution in all MS-based strategies, we wondered about the relevance of high mass accuracy of mass spectrometers for the identification of proteins, peptides and metabolites, and whether there is still a need for even higher accuracy, an often used selling point in the competitive MS instrumentation market. To this end, we compared the number of observable different masses of proteins, peptides and metabolites at different theoretical mass accuracies (between 0.25 ppb and 7000 ppm, based on the lowest mass in the compound class). For display purposes, these were then classified into mass bins (Fig.3A–C).

Accordingly, a mass accuracy of <7.1 ppm (which equals 0.01 Da in the 1.4 kDa protein LST1) would be sufficient to distinguish the masses of 35 857 of 35 956 unique proteins (99.7%) (Fig.3A). Even <71 ppm would cover 98.3% of the human proteome, a mass accuracy that is achievable even by previous generation MS instruments. However, the presence of PTMs was not considered in these calculations. PTMs will significantly increase the number of different masses and also mass redundancy, since many proteins can be modified in different ways on a multitude of sites. Furthermore, multiple charge states add another layer of complexity to the data, which is still analytically challenging, especially in protein mixtures. Until recently ‘Top-Down’ proteomics was limited to the identification of protein numbers in the low hundreds. The work of Tran et al. 32 exemplifies the extensive work necessary to push these numbers towards the identification of 1000 gene products and more. While the information gain about splice forms, PTMs and endogenous protein cleavages compared to ‘Bottom-Up’ proteomics is certainly significant, the analytical and biochemical investment is a major factor and far from routine.

In a tryptic digest, a mass accuracy of <2.5 ppm (which equals 0.001 Da in a peptide of 400 Da) could distinguish 253 489 of the 279 002 (90.9%) different masses (without considering PTMs), while <0.25 ppm would cover 99.9% of all different peptide masses (Fig.3C). Such a mass accuracy can be provided by modern Trap, ICR and TOF-MS instruments with software-aided recalibration of the data 113. Even though we expect less mass redundancy with other proteolytic enzymes, a mass accuracy of <0.25 ppm is also needed to distinguish the proteolytic peptides by their mass when using alternatives to trypsin (Supporting Information Table 1). On the peptide level, PTMs add to the number of unique masses and mass redundancy, yet the impact on the depicted numbers would be marginal compared to proteins since one PTM would only change the mass of a tryptic fragment of a protein while other peptides remain unchanged. Commonly occurring missed cleavages, if they are not caused by the presence of a neighbouring PTM, can be predicted 34 and therefore would reduce the sample complexity and increase the number of longer peptides with unique masses. Nevertheless, the identification of a peptide sequence, only based on the detection of a precursor mass in a tryptic digest, would work for 197 709 unique masses.

Manufacturers of mass spectrometers often claim that high mass accuracy is the key to the identification of proteins, peptides and metabolites. In the case of metabolites, matching highly accurate mass, retention time and fragmentation spectrum with a synthesised standard is considered the gold standard for the identification of a metabolite 114 – especially with GC-MS instruments (since chromatographic reproducibility is superior to LC-MS workflows). Considering the METLIN database, we found that all 64 092 reported compounds were represented by 17 058 different masses (Fig.3C) and only 8828 entries (13.8% as compared to 49.7% of proteins in the human tryptic peptidome) have a unique mass. If the mass spectrometer achieves a mass accuracy of <7 ppm (based on the mass of methane, 16.0313 Da), we can still distinguish between 16 933 masses (99.3%). Even with <70 ppm accuracy, 97.9% of all masses would be detectable as separate entities given absolute resolution or a complete front-end separation of the sample. Considering the front-end separation of the analyte and the low number of small molecules in a human serum sample (4651 human metabolites 115 and other detectable small molecules such as endogenous peptides, drugs and their degradation products as compared to a tryptic digest of the human proteome), the mass accuracy of today's mass spectrometers is more than adequate. However, high accuracy in mass measurements can often narrow down combinatorial possibilities of matching molecular formulae, a feature that is often used in the identification process of small compounds/metabolites.

## 7 Data analysis

For the analysis of proteomic MS data there are a few established software workflows available that are, similarly to rather uniform sample preparation protocols, widely used in proteomics research laboratories. Peptides are identified by the comparison of the submitted MS/MS spectra extracted from raw data with theoretical fragmentation patterns derived from protein sequences. MS/MS spectra of peptides are typically rich in diagnostic ions, and the matching of fragment masses to predicted mass tables is only a computational problem as this workflow is nowadays a fully automated and fast process in silico, so that even highly complex data can be searched within minutes (Mascot 116, MaxQuant 117 and others). The number of spectra identified in a given sample can exceed 60% of total acquired spectra, even when repetitive selection of a peptide for MS/MS analysis is minimised. The validity of identifications is estimated with false discovery rates or probability scores (reviewed by 118). The entire analysis, including the use of a variety of different search algorithms, can be automated and streamlined 119, resulting in peptide/protein tables, which can then be used for a systematic analysis (i.e. ingenuity pathway analysis, KEGGS, DAVID, STRING, Metacore and others) to reveal expression changes, protein–protein interactions and the presence of PTMs.

In contrast, the identification of metabolites in untargeted studies is fundamentally different to the identification of proteins (Fig.1). Spectral libraries such as METLIN contain information about mass and structure of small molecules, although MS/MS spectra are available for only a (increasing) share of the small molecules in the database. This limitation impedes the easy and quick identification of small molecules in a sample. Even the basic workflow that can be adapted from a proteomics MS machine setup (positive ionization and a RP column) yields hundreds of molecular features from a blood sample within minutes of data acquisition 24. After correcting the data for adducts, neutral losses and multimers, only a minority of detected masses can be matched to a database entry, or more commonly to several possible molecular formulas. Therefore, identical matching mass does not necessarily mean identification, and as we have shown in Fig.2 and Supporting Information Fig. 1, the mass redundancy in organic molecules will result in a variety of different candidates for one detected mass. A definite manual identification can only be achieved by a matched MS/MS spectrum and/or by another compound-specific property such as chromatographic behaviour (retention time), which is then compared to a synthesised standard compound.

The presence of stable isotopes in natural compounds (^13^C, ^15^N, ^18^O, ^34^S, etc.) can be exploited for the assignment of a detected signal to a group of compounds with a different elemental composition. If the isotopic peaks of a compound can be resolved by the mass spectrometer, its isotopic distribution can be used to identify the elemental composition by matching to a theoretical isotope pattern 24. This process can be automated and used for scoring of a potential identification of a compound (i.e. using Mass Profiler Professional Software (Agilent)). While the structural information, which can be provided by the isotopic peak pattern, is used for metabolite analysis routinely, the proteomics field neglected this source of information until recently. Miladinovic et al. reported the determination of the isotopic fine structure in peptides using a current high magnetic field FT-ICR with a resolving power of ∼2 000 000 120. Acquired MS spectra could resolve the isotopic envelope of the individual isotopic peaks and provide structural information that can be used to improve peptide identification.

In principle, quantitative analysis in metabolomic experiments is very similar to the label-free quantitation approaches based on extracted ion chromatograms in proteomic workflows. Feature alignment and detection is followed by quantitation and then perhaps identification of a compound. However, the tendency of small organic molecules to form multimers or adducts (i.e. sodium or ammonium) needs to be considered, and detected masses and their intensities deconvoluted before quantitation and statistical evaluation. While in proteomics a variety of highly functional software for identification and quantitation of peptides/proteins is available, the metabolomics field has seen a recent boost of new and very often freely available software (http://www.metabolomicssociety.org/software, XCMS (http://metlin.scripps.edu/xcms/) and http://fiehnlab.ucdavis.edu/staff/kind/Metabolomics/Peak_Alignment/). Also, typically instrument vendors and software developers offer commercial software solutions (i.e. Agilent's Mass Profile Pro or nonlinear dynamics’ Progenesis CoMet). The number of available MS/MS spectra for small molecules is limited but increasing. Compared to feature-rich peptide-derived MS/MS spectra, small molecules generate far less diagnostic ions that can be used for identification. Also, MS/MS spectra are not easy to predict and require de novo interpretation. This can be aided by web-based tools such as MetFrag 121, which can match detected fragment masses to in silico generated molecule fragments and assist in the identification of metabolites.

## 8 Conclusions

This review is primarily addressed at proteomics laboratories that are exposed to a growing demand for the analysis of small molecules and metabolites. Measuring metabolites using a proteomics MS workflow, based on RP LC MS/MS, can be performed with little to no investment in additional hardware. However, a metabolomics researcher may argue that analytical workflows used for proteomics cannot observe the whole metabolome (-OMICS) in a sample. While this is certainly true, it does not mean that the admittedly limited compatibility of an organism's metabolome with a RP- or HILIC-based nano-flow chromatography invalidates the use of nLC-MS. Any other analytical workflow for metabolomics will have the same principal limitations due to the vast chemical diversity in metabolite samples. Data generated by LC-MS-based systems can complement data from instrumentation commonly used for metabolomics such as normal-phase NMR, GC-MS, etc. by detecting a subset of metabolites. We argue that especially for untargeted discovery experiments, instrumentation usually employed in proteomics laboratories can be used as a starting point for metabolomic studies.

While the principles of data generation between meta-bolome and proteome samples using nLC-MS systems are very similar, the qualitative data analysis is very different. With the exception of SELDI 122, the central part of a proteomic workflow is always the identification of the analyte. This is in contrast to metabolomics screening experiments where the identification is not required to generate a molecule profile that may be specific for an experimental condition or disease (see Fig.1, highlighted nodes). Accurate mass is a very important feature in proteomics. This is almost certainly less critical for (human) metabolites, since the vast majority of molecules in METLIN (89%) have a mass below 1000 Da, and the mass accuracy of modern mass spectrometers is sufficient to distinguish between most masses (99.3% 7 ppm, Fig. 3B). Proteomic researchers are used to assign up to 60% of detected peptides even in complex samples to a peptide sequence. Of the thousands of detected small molecules in the serum metabolome, only a very small fraction will be readily mass-matched to a set of compounds in a relevant database. For a fraction of those compounds only there will be MS/MS spectra available.

Our proposal to employ the instrumentation and expertise for peptide identification – readily available in proteomics laboratories – to identify and quantify metabolites from complex samples has its limitations, especially if a comprehensive mapping of a metabolome is desired. A laboratory, in which proteomic and metabolomic workflows are employed side by side to overcome the specific limitations of each individual technology, would require high financial commitment and widely spread expertise. The use of nLC-MS-based instrumentation can therefore be a valid compromise, enabling the identification and quantitation of a subset of a metabolome, which can be separated and ionised on a platform normally used for peptide identification. We described ways to expand the number of detectable compounds by employing alternative chromatographic methodologies such as HILIC or alternative ionization techniques, which are employed relatively easy and with little financial effort. We have also pointed out that some analytical challenges (i.e. high mass redundancy), which are very familiar to proteomic researchers, are also apparent when analysing small molecules. Interestingly, technical limitations such as mass accuracy appear less of an issue than originally thought, especially because exact mass matching is often not enough and other parameters are clearly required for identification. While the lack of characterization of the metabolome is nothing new to metabolomics specialists, a proteomic researcher undertaking first steps in metabolomics may need to think out of the black box, which is the unity of LC-MS instrument and automated data analysis. For metabolomics studies, the major challenges will remain the adequate identification and characterization of measured molecular compounds. Expertise from both disciplines can be complementary in the advancement of methodologies, in particular for integrative analyses of complex biological samples.

## Abbreviations

APCI: atmospheric pressure chemical ionization
APPI: atmospheric pressure photoionization
HILIC: hydrophilic interaction chromatography
