# Supplementary material for: Two birds with one stone: Doing metabolomics with your proteomics kit
Source: Proteomics. 2013 Nov 21;13(23-24):3371–86. doi: 10.1002/pmic.201300192 (PMC4265265; doi:10.1002/pmic.201300192)

**Supplementary data**

**Figure S1:** Distribution of unique and redundant masses in the human proteome, tryptic peptidome and metabolome (METLIN database). The number of unique masses in the three analytes were counted and depicted as share of unique compounds (proteins, tryptic peptides, METLIN entries). The determination of the mass of a unique compound would facilitate its identification. The METLIN metabolome has the highest mass redundancy, resulting in the requirement of orthogonal data for the identification of most compounds. PTMs were omitted for Proteins and peptides but would result in higher mass redundancy than depicted
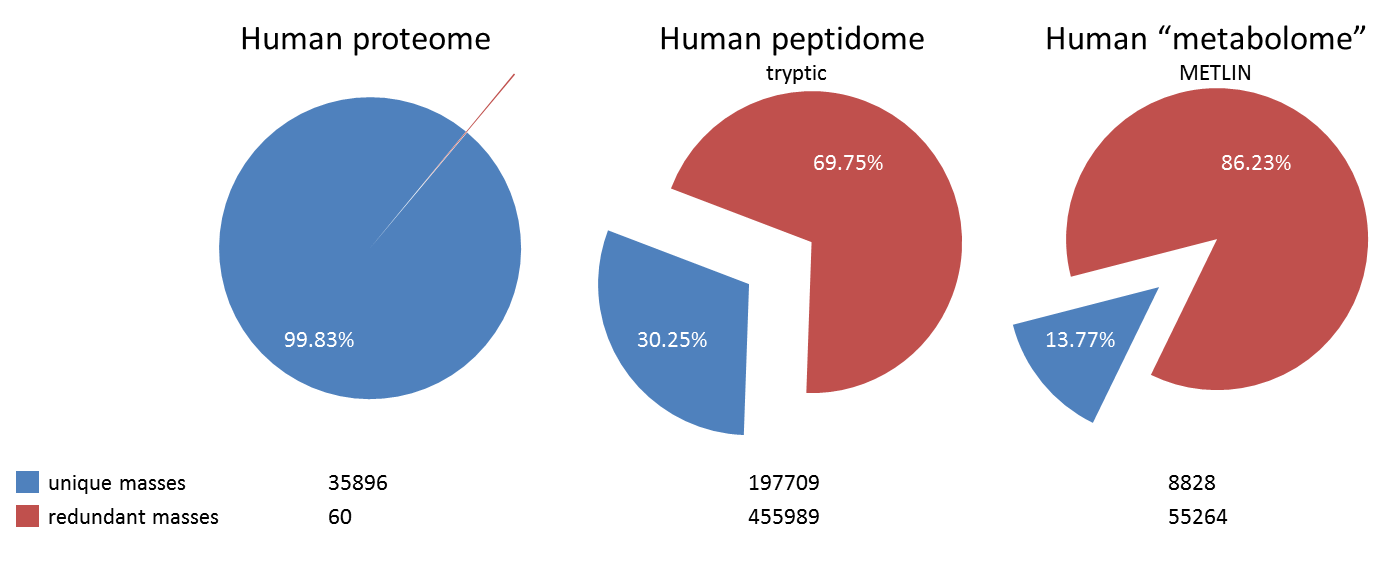


**Figure S2: Sample fractionation by Chloroform-Methanol Precipitation** . The aqueous phase can be analysed for hydrophilic compounds and endogenous peptides using reversed phase chromatography coupled mass spectrometry. The protein interphase can be further processed to be analysed in a proteomic workflow while the organic phase is not compatible with standard LC-MS equipment. MALDI or GC-MS instrumentation can be used to detect hydrophobic compounds in the water-insoluble fraction.


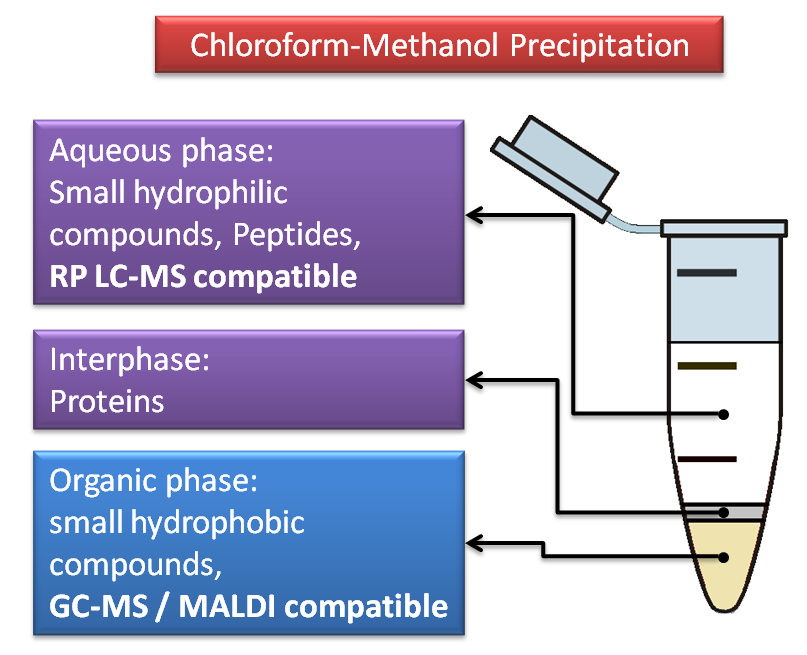


**Figure S3:** Display of the number of different peptide precursor masses derived from an *in silico* digestion of human proteins present in the SwissProt (UniKProt) database with various proteolytic enzymes and their separation based on mass.


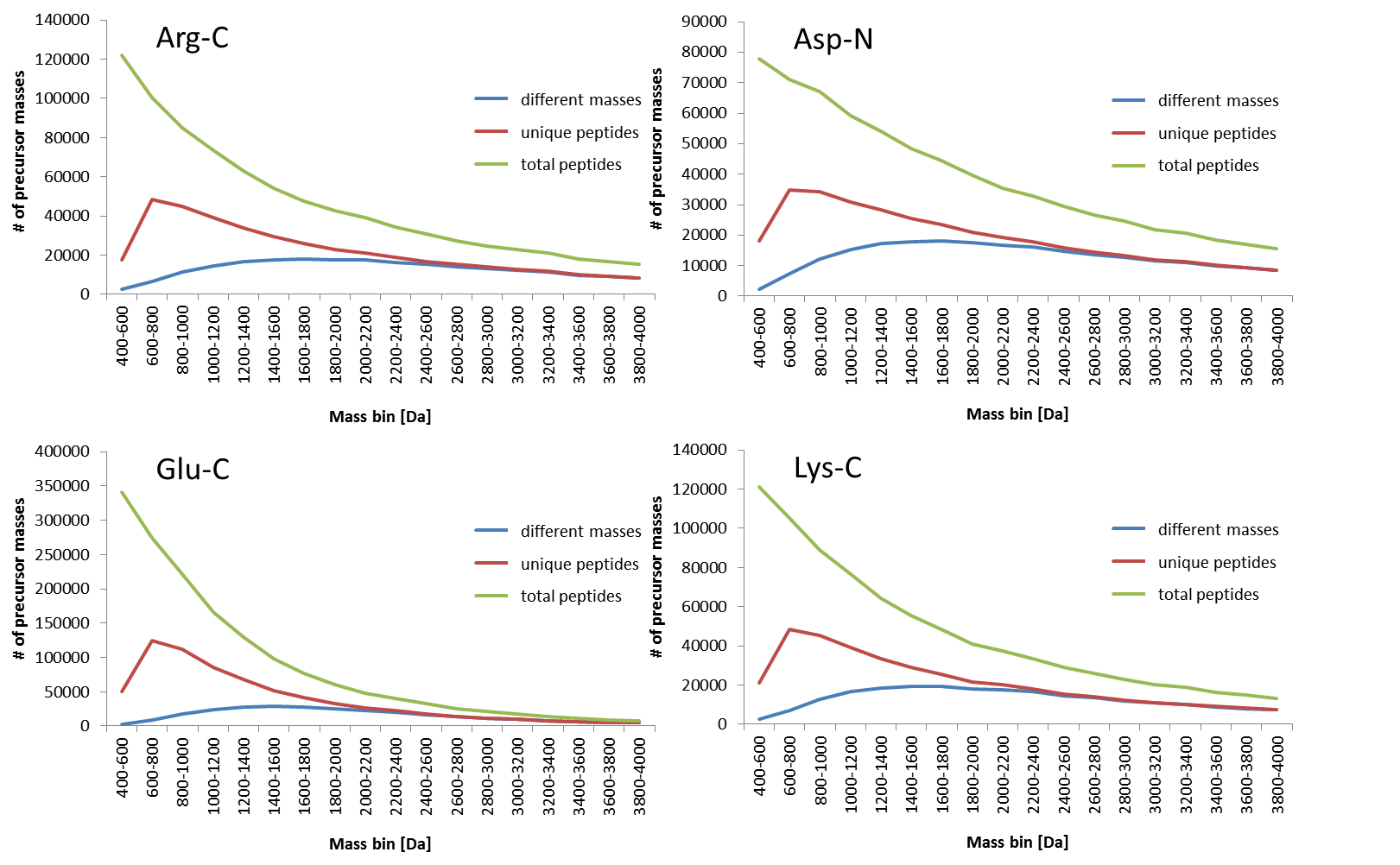


**Table S1:** Number of distinguishable masses in in the human proteome after *in silico* digestion with various proteolytic enzymes. The calculations are based on different mass accuracies and requiring absolute resolution and/or frontend separation. A mass accuracy of 0.25ppm will allow distinguishing between the vast majorities of different peptide masses within the calculated peptide populations.


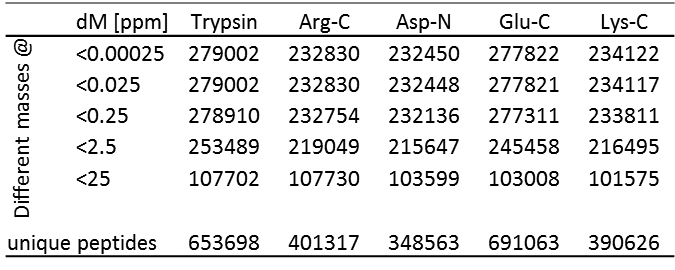


**Table S2:** Calculation of mass redundancy in enzymatic digests of the human proteome using different proteolytic enzymes.


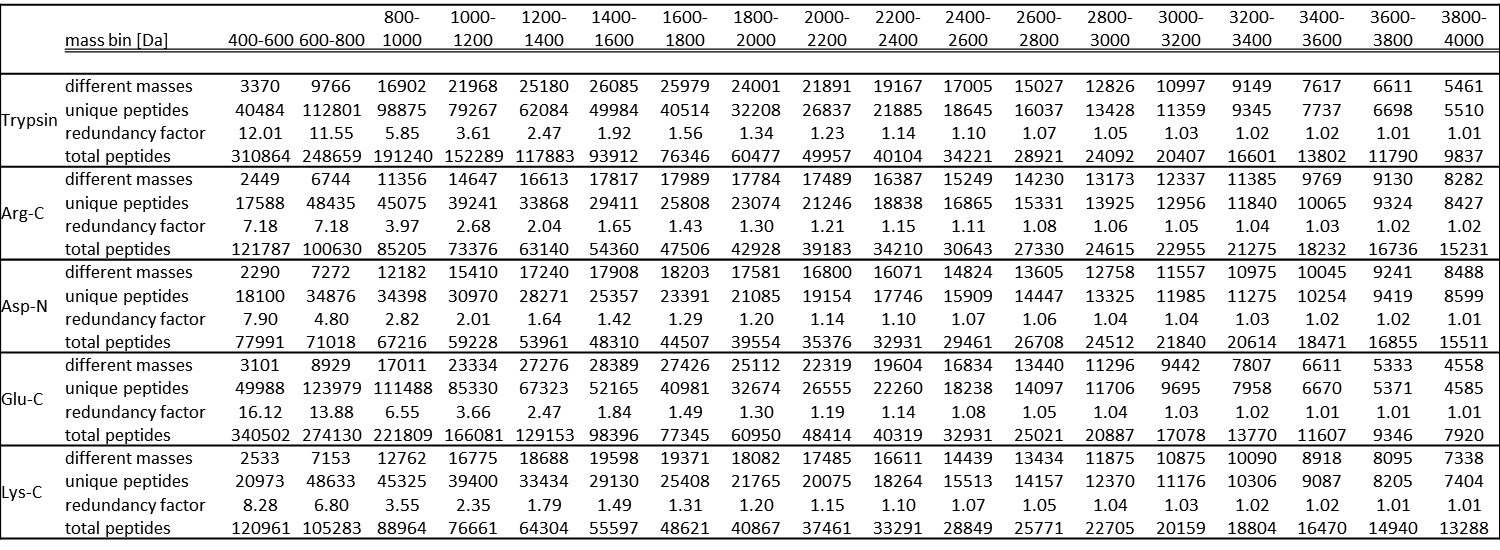

Supplement: Supplementary file 1 [file pmic0013-3371-SD1.doc]
